# Multi-Level Sensing Technologies in Landslide Research—Hrvatska Kostajnica Case Study, Croatia

**DOI:** 10.3390/s22010177

**Published:** 2021-12-28

**Authors:** Laszlo Podolszki, Ivan Kosović, Tomislav Novosel, Tomislav Kurečić

**Affiliations:** Croatian Geological Survey, Sachsova 2, 10000 Zagreb, Croatia; ivan.kosovic@hgi-cgs.hr (I.K.); tomislav.novosel@hgi-cgs.hr (T.N.); tomislav.kurecic@hgi-cgs.hr (T.K.)

**Keywords:** multi-level remote sensing of soil and rock, different level data sets analysis, model development, landslide, case study

## Abstract

In March 2018, a landslide in Hrvatska Kostajnica completely destroyed multiple households. The damage was extensive, and lives were endangered. The question remains: Can it happen again? To enhance the knowledge and understanding of the soil and rock behaviour before, during, and after this geo-hazard event, multi-level sensing technologies in landslide research were applied. Day after the event field mapping and unmanned aerial vehicle (UAV) data were collected with the inspection of available orthophoto and “geo” data. For the landslide, a new geological column was developed with mineralogical and geochemical analyses. The application of differential interferometric synthetic aperture radar (DInSAR) for detecting ground surface displacement was undertaken in order to determine pre-failure behaviour and to give indications about post-failure deformations. In 2020, electrical resistivity tomography (ERT) in the landslide body was undertaken to determine the depth of the landslide surface, and in 2021 ERT measurements in the vicinity of the landslide area were performed to obtain undisturbed material properties. Moreover, in 2021, detailed light detection and ranging (LIDAR) data were acquired for the area. All these different level data sets are being analyzed in order to develop a reliable landslide model as a first step towards answering the aforementioned question. Based on applied multi-level sensing technologies and acquired data, the landslide model is taking shape. However, further detailed research is still recommended.

## 1. Introduction

**Figure 1:**
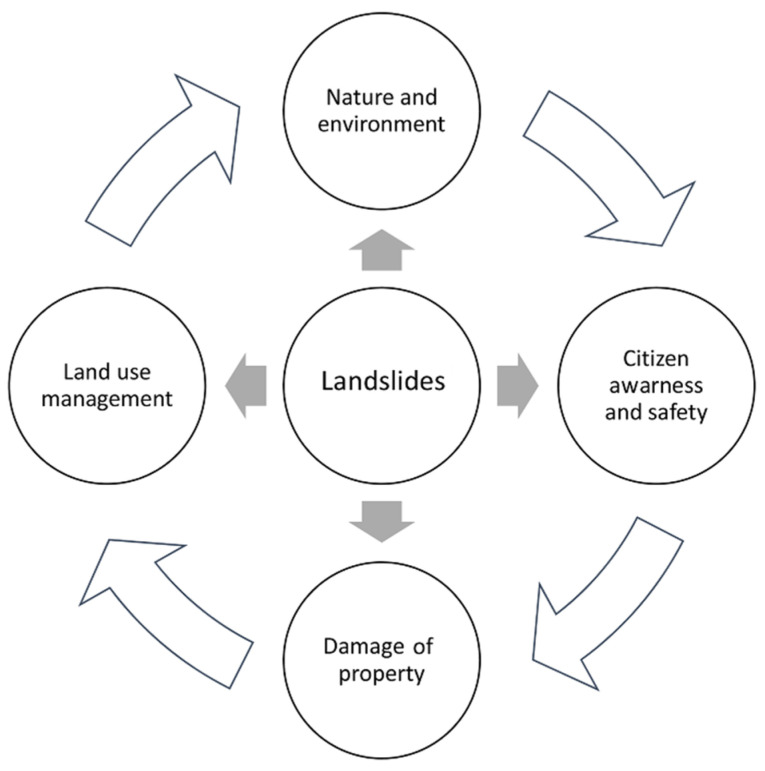
Landslides affect our environment and land use management and they can present danger to properties and safety.

Geohazards are constant threat to human activity in the ever-changing environment [1,2]. Due to climate changes, some natural extremes are even more emphasized, including rapid temperature changes, flooding, and high amounts of precipitation in a short period, all of which are events that can affect slope stability in general [3,4]. As a consequence of this trend, new landslides occur and old ones are being reactivated [5,6]. In combination with anthropogenic activity, landslides affect our surroundings greatly (Figure 1) and the need for future landslide risk reduction is getting more and more important [7,8,9]. In risk reduction, the prediction of the behavior of soil and rock during and after extreme events is a key but also complex task [10]. In this process, different (remote) sensing technologies with optimal parameters input [11] can acquire much needed data and enhance the knowledge and understanding of the site-specific problem [12,13,14,15] and in some cases even offer solutions [12,13,14], help in early warning [15] or in disaster control [16,17].

In the literature, different variations and combinations of remote sensing data have been utilized in different landslide case studies, mainly depending on the available data, technology, site specific characteristics, and goals, e.g., (i) with a combination of monitoring techniques (satellite imagery, digital elevation models, robotized total stations) insights were obtained for the geo-mechanical modelling of earthflows [18]; (ii) landslide dynamics was analyzed based on data acquired by unmanned aerial vehicle [19]; (iii) light detection and ranging and unmanned aerial vehicle data were combined to evaluate the geomorphological features of a landslide [20]; (iv) morphological changes of the earthflow were assessed, comparing multi-source and multi-temporal data (aerial photographs, satellite images, light detection and ranging, and unmanned aerial vehicle data) [21]. Generally, these data sets can be considered as “2D (planar) remote sensing data sets” and for the area of research (Hrvatska Kostajnica landslide) not only were “2D (planar) remote sensing data sets” considered (orthophoto, satellite imagery, unmanned aerial vehicle and light detection and ranging data), but an insight into the third dimension (depth) was provided by geophysical profiling, i.e., “3D (vertical) remote sensing data sets” were used.

Hrvatska Kostajnica area, in northern Croatia, has a history of landslides and damage to buildings and infrastructure caused by landslides. However, available landslide data for this area are relatively sparse and no landslide inventory or typical landslide model exists. The aim of this research was to develop such a landslide model by integrating new approaches in geohazard research by multi-level data analysis: different remote landslide data were acquired and existing and new geological data related to the study area were analyzed and interpreted.

## 2. Materials and Methods

### 2.1. Study Area

**Figure 2:**
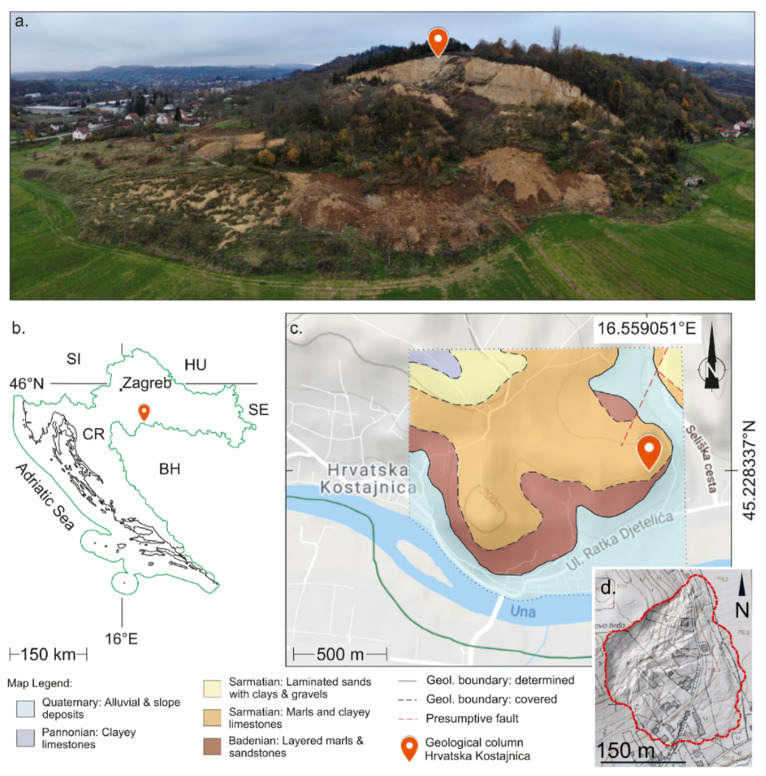
Study area: (**a**) landslide panorama in Hrvatska Kostajnica—on the south-eastern slope of Kubarnovo brdo, photo taken by unmanned aerial vehicle (UAV), by Croatian Geological Survey (CGS); (**b**) landslide location is in Croatia, near the border with Bosnia and Herzegovina; (**c**) basic geological data [23,24]; and (**d**) detail from topographic map (before landslide) overlaid with landslide area and detailed hillshade DEM (after landslide) and houses within landslide area: destroyed and damaged. Landslide area was defined by Croatian Geological Survey (CGS).

**Figure 3:**
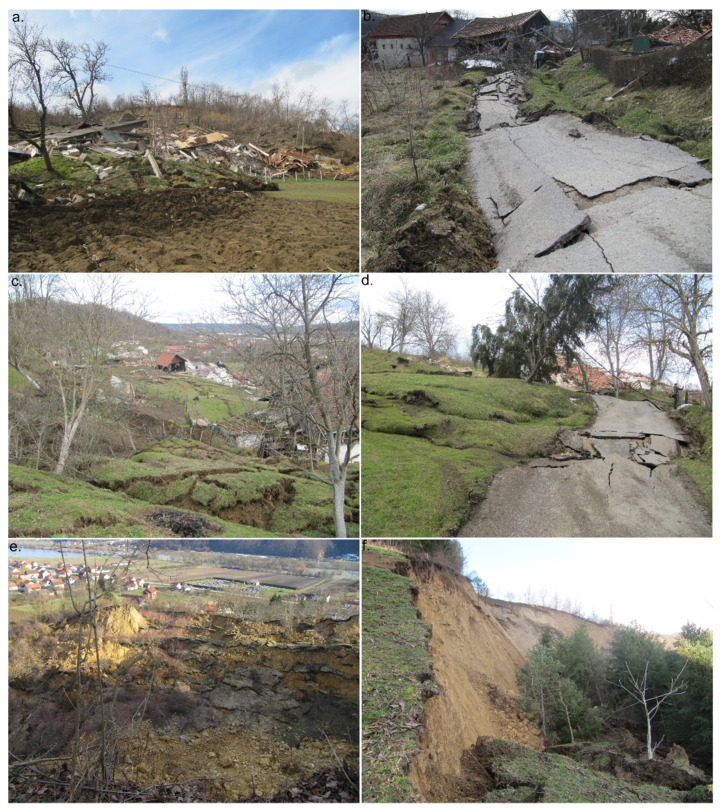
Hrvatska Kostajnica landslide damages: (**a**) completely destroyed houses; (**b**) completely destroyed road and damaged objects; (**c**) cracks and heavy terrain deformations; (**d**) flow like deformations on terrain surface; (**e**) view from main scarp toward flooding Una River; (**f**) landslide main scarp—almost 30 m at the highest point (photos by Croatian Geological Survey (CGS), day after the event, 14 March 2018).

The study area is in Croatia in the Sisačko-Moslavina County in the city of Hrvatska Kostajnica (Figure 2a), near Una River, which is the border with Bosnia and Herzegovina (Figure 2b). The city area is 53 km^2^ and the population is ~3000. Una River is situated ~400 m south from the Kubarnovo brdo where on 13 March 2018 the landslide occurred (Figure 2a–d). Una River is ~100 m above sea level, while Kubarnovo brdo peak is 188 m above sea level. Landslide is mainly rotational (only in some smaller parts can be considered as composite, a combination of types: rotational and translational slide and flow) [10,22], with area of ~300 × 300 m and maximum of ~61 m height difference: the highest part of the landslide (crown) is at 175 m above sea level while the lowest part of the landslide (toe) is 114 m above sea level. In combination with geological setting (Figure 2c), rapid temperature change (from below 0° to above zero which caused ~80 cm of snow cover to melt), period of rain, and Una River flooding, slope failure occurred (Figure 2d) and completely destroyed multiple households. The damage was extensive, and lives were endangered (Figure 3a–f).

Geographically, the study area is located on the south-eastern slope of Kubarnovo brdo, a hilly area (Figure 2a). The landslide toe part is on the flat area near Kostjničica stream situated only 100 m east from the landslide, and ~400 m south is Una River. Geologically [23], within Quaternary alluvial and silty flood sediments slope deposits are also present. The landslide occurred in Sarmatian marls and clayey limestones and Badenian layered marls and sandstones (Figure 2c) [24]. In terms of engineering geology [25,26] there is soil on the surface in the area. The thickness of soil varies but it is generally thinner than 0.5 m. At the main scarp area, the soil layer thickness is between 0.3 and 0.7 m. Below the soil layer, there is a layer of diluvial sediments which are represented by clays and clayish marls up to 3 m thickness (estimated). These sediments are weathered, and the weathered zone can reach up to 5 m of depth (estimated). This zone of weathered bedrock is generally below the stretch of the diluvial sediments. These weathered materials are (mainly) composed of sandy–silty-clays. Below the weathered zone bedrock follows, which is represented by clayish marls, silty marls, as well as layers of sandstones and weathered clayish limestones. The landslide has affected the soil layer, weathered zone, and bedrock, so the material characteristics vary in a wide range from soils to rocks. The actual study area covers ~500 × 500 m and it is somewhat wider than the landslide area.

### 2.2. Data Sets

Data sets used in this research consisted of reviewed existing and interpreted new data in different forms: maps, remote sensing data, field research, and laboratory data with associated analysis and re-interpretation. The main used data sets are listed in Table 1, but the main focus was on acquired multi-level sensing data sets, such as orthophoto data, unmanned aerial vehicle (UAV) data, synthetic aperture radar (SAR) data, light detection and ranging (LIDAR) data, and geophysical measurements, i.e., electrical resistivity tomography (ERT) data.

### 2.3. “Historical” Geological and Landslide Data Review

**Figure 4:**
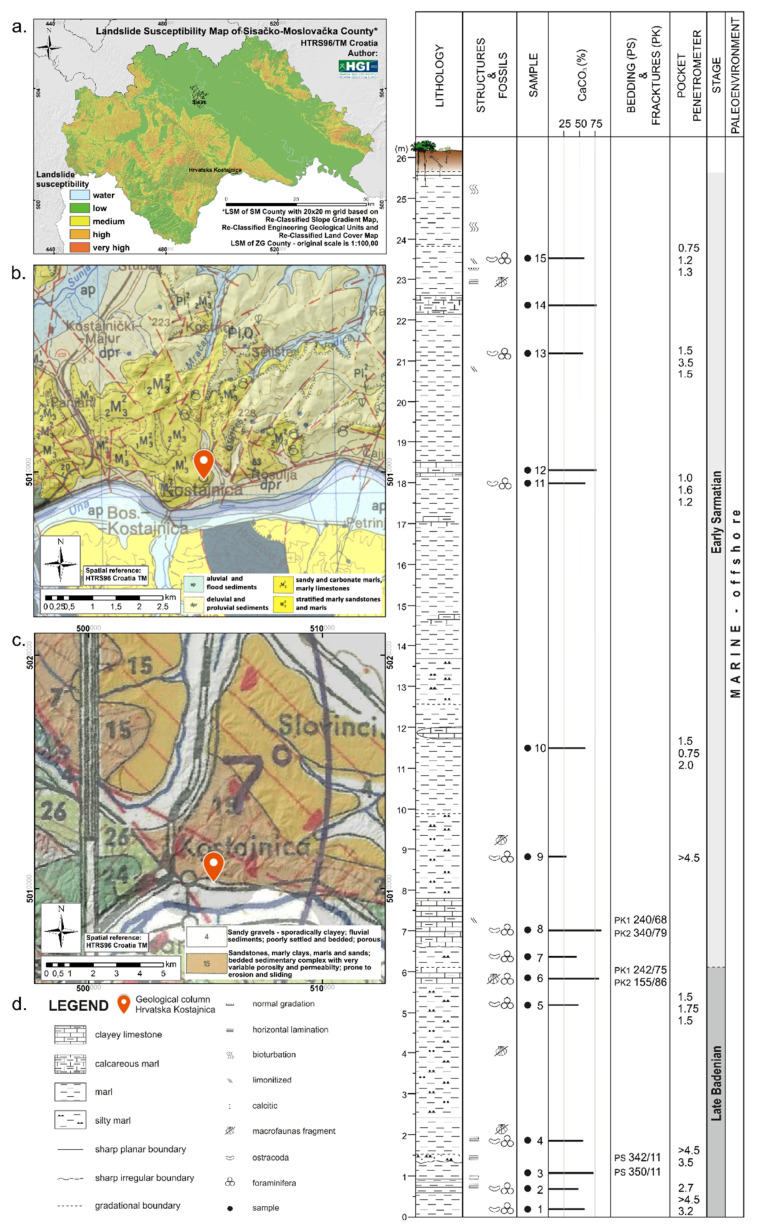
Study area “zoom in”: (**a**) landslide susceptibility Map of Sisačko-Moslavina County [4]; (**b**) detail from the Basic geological map [23]; (**c**) detail from the Engineering Geological Map [28]; and (**d**) the developed detailed geological column for Hrvatska Kostajnica landslide, modified after [24].

Available geological and landslide data were reviewed, mostly in the forms of existing guides and maps (Table 1, Figure 4a–d). Hrvatska Kostajnica is in the continental part of Croatia, where soils are commonly developed on the surface of the different types of sediments and slides in soils frequently occur (Figure 4a) [4].

This is also reflected in the Re-Classified Lithological Map of Croatia, where clayey materials and alluvial/diluvial deposits are depicted in the study area [6]. The 1:100,000 scale Basic geological map (Figure 4b) [23] and the 1:100,000 scale Geological Map of Sisačko-Moslavina County [29] indicate that Badenian (conglomerates, sandstones, marls, limestones) and Sarmatian (sandstones, gravels, sands, marls) deposits are present in the area with diluvial and proluvial Holocene sediments (sands, silts). Near the Una River, alluvial Holocene sediments (sands, gravels, silts, clays) are common [24]. In the 1:100,000 scale basic geological map, the presumptive fault locations are also marked (red dashed lines, Figure 4b) [23].

On the Landslide Susceptibility Map of Croatia, the wider area is generally depicted as prone to landslides [27], as on the 1:100,000 scale Landslide Susceptibility Map of Sisačko-Moslavina County (Figure 4a) [4] and on the 1:500,000 scale Engineering Geological Map of Yugoslavia, on which active landslide locations are marked with red semi circles (Figure 4c) [28]. The detail from the 1:500,000 scale Engineering Geological Map indicates that two units are present in the study area: (i) sandstones, marly clays, marls, and sands; Upper Neogene lacustrine bedded sedimentary complex with very variable porosity and permeability, prone to erosion and sliding (brown on map); and (ii) sandy gravels, sporadically clayey; Pleistocene–Holocene fluvial sediments, mostly covered with loam, poorly settled and bedded, rather porous (white on map). The topographic maps (1:5000 from NGA and the one developed from LIDAR data) in combination with developed landslide inventory for study area provides a good idea about the spatial distribution of landslides in this area (shown in detail in Figure 2d).

A detailed 1:5000 geological map for the Hrvatska Kostajnica landslide area is being developed by the Croatian Geological Survey while a detailed geological column for the Hrvatska Kostajnica landslide has already been developed (Figure 4d) and modified within this research [24]. All of the available “geo data” provide a good basis for quality terrain surface model development (2D), but some questions remain concerning sediment distribution and actual sliding surfaces(s) depth remains (3D). Some “3D” answers were given by the developed geological column and performed ERT measurements.

### 2.4. Remote Sensing Data and Methods Used

In this research multi-level remote sensing technologies and data were used: DInSAR, orthophotos, UAV, LIDAR, and ERT.

For the Hrvatska Kostajnica area the method of SBAS-DInSAR was employed to obtain time series displacement [26,30]. The synthetic aperture radar (SAR) is an active remote sensing imaging system. Interferometric SAR (InSAR) is a method for taking the signal phase changes (interference) from two scenes of SAR data, which are observed in the same area during different periods, by exploiting repeated orbits of the satellite [31]. Differential interferometric SAR (DInSAR) is the commonly used for the production of interferograms from which the topographic influence has been removed. The advantage of DInSAR is that it can provide centimeter-scale displacements of the surface of the Earth (i.e., changes in length between the radar and the ground surface) over vast areas of thousands of square kilometers with a spatial resolution of 3–30 m [32]. The displacement measured by DInSAR is one dimensional, along the satellite’s line of sight (LOS), and referred to as LOS displacement.

Orthophotos from National Geodetic Administration of Croatia (NGA) were reviewed as a source of historical (from 1968), pre-failure (from 2014–2016), and post-failure data (from 2017–2018 and 2020), Table 1 [12,33].

The Croatian Geological Survey performed field mapping and unmanned aerial vehicle (UAV) recording on 14 March 2018 (day after the event) [25]. Obtained orthophotos have 5 × 5 cm pixel size with margin of error ±10 cm (Table 1). From them, a 3D landslide area spatial model was also developed.

Based on aerial LIDAR scanning from spring 2021 with a point cloud density of 20 points per m^2^ and with accuracy of ±10 cm, high resolution DEMs (0.5 × 0.5 m cell size) were created and interpreted for the Hrvatska Kostajnica area (Table 1). During LIDAR scanning, high resolution orthophots (10 × 10 cm pixel size) were also acquired.

The electrical resistivity tomography (ERT) method as well as the other near-surface geophysical methods are widely used for the assessment and forecasting of landslide processes [34,35,36]. The purpose of electrical surveys is to determine the subsurface resistivity distribution by making measurements at the ground surface [37,38,39,40]. From these measurements, apparent resistivity, geometry of landslide, zones of discontinuities, and the depth of the slide surface can be acquired/assessed. As changes in resistance depend on changes in humidity, as well as differences between geological units/properties, this method is very useful for identifying moisture or seepage zones, which is very important for the study of slide surface(s) of landslide at Kurbanovo brdo, Hrvatska Kostajnica (Table 1).

### 2.5. Laboratory Data Review

Laboratory analysis for the Hrvatska Kostajnica landslide/geological column development included mineralogical and geochemical analyses: X-ray powder diffraction (XRPD, 6 samples), chemical analysis of major and trace elements (7 samples) and measurement of CaCO3 using Scheiblers calcimeter (15 samples) accompanied by paleontological analysis (calcareous nannofossil, palynological, foraminiferal, and ostracod analysis). A detailed laboratory analysis review is provided by Grizelj et al. [24] with the following main conclusions: the geological Hrvatska Kostajnica column represents continuous sedimentation from the late Badenian to the early Sarmatian and the mineral composition of pelitic sedimentary rocks is common for Middle Miocene deposits. The bulk analysis of marls shows that the main mineral components are calcite and clay minerals (i.e., smectite, ilite, kaolinite, vermiculite) with some of them with high shrink-swell capacity. All of the analyzed samples were gathered at the landslide head scarp (26 m height) with the single rope technique by one of the authors (T. Kurečić).

## 3. Results

### 3.1. DInSar Analysis Results

This method implements a simple combination of interferograms produced by multiple SAR data pairs. The SAR data pairs are characterized by a small spatial baseline and temporal baseline. The idea was to detect pre-failure movements or post-failure subsidence on the area (150 scenes from Sentinel 1 A and B for the period of 1 December 2014–13 June 2020 were analyzed), Table 1.

Only a short overview is described here as the undertaken DInSAR analysis was presented at the Geotechnical Conference in Omiš, Croatia, 2019 [26] and it is supplemented by PhD research from 2020 [30] as one of the case studies for that research.

The spatial distribution of LOS displacement showed the following: (i) there is no remarkable LOS displacement around the landslide area, although displacement slightly increased around the active landslide area, and (ii) the large displacement(s) are found only around the cropping/farming area, near Una River.

DInSAR could not detect the large LOS displacement induced by the landslide occurring on 13 March 2018. Only 2 cm displacement was detected in the center of landslide area, although the real landslide displacement is more than a few meters.

Essentially, DInSAR cannot measure exactly a displacement over a half wave length of the SAR signal (2.8 cm in the case of C-band used by Sentinel-1) for one analysis. This is a common disadvantage of DInSAR (in some cases).

### 3.2. Orthophotos Analysis Results

**Figure 5:**
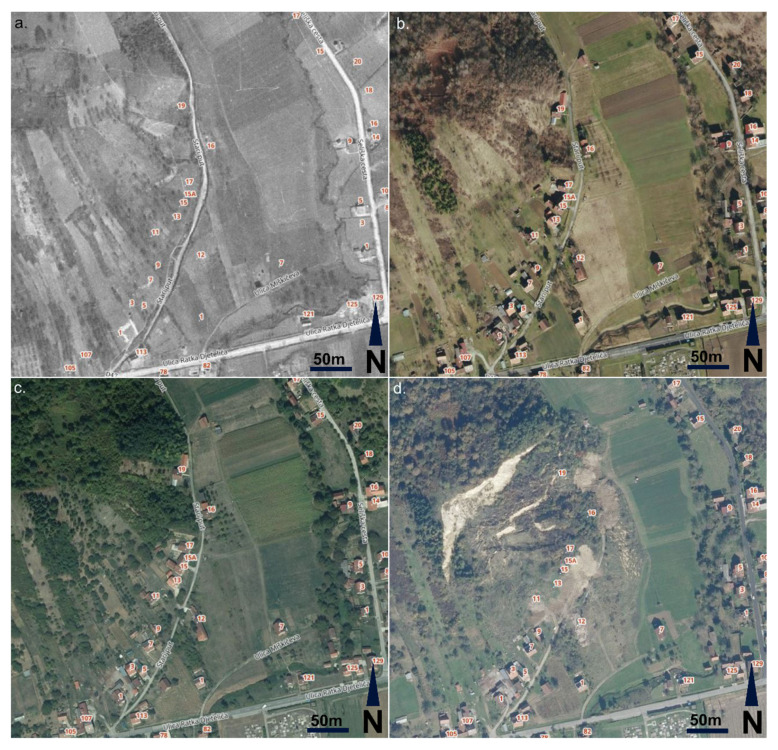
Hrvatska Kostajnica landslide area orthophotos from National Geodetic Administration of Croatia (NGA): (**a**) from 1968—historical data; (**b**) from 2016—pre-failure data; (**c**) from 2018—(recent) pre-failure data; and (**d**) from 2020—post-failure data.

**Figure 6:**
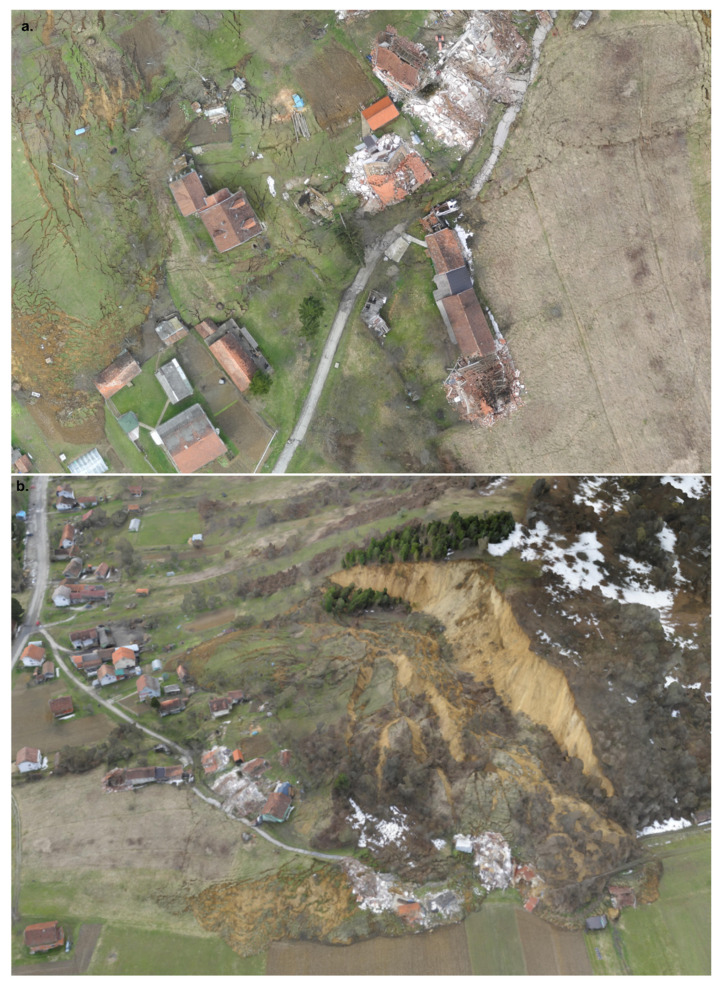
Hrvatska Kostajnica landslide area on 14 March 2018 (day after the event)—UAV data collected, analysed and developed by CGS: (**a**) detailed orthophoto data; and (**b**) 3D landslide area spatial model.

Available orthophotos were reviewed as a source of land use change records (Figure 6a–d) [12,32]. In the historical photo from 1968, the landslide area is cropland without houses/objects (Figure 5a). On the “recent” photos from 2016 (Figure 5b) and 2018 (Figure 5c), the pre-failure situation can be observed: houses (1–19 house numbers), croplands, and woods. On the post-failure photo from 2020 (Figure 5d), it is visible that houses 11–19 are removed (as they were destroyed or heavily damaged by landslide) and the croplands are destroyed. Houses 7 and 9 are damaged while houses 1, 3, and 5 are still endangered. In all figures (Figure 5a–d), the house numbers at Stari put Street are marked as reference. Stari put Street was completely destroyed by the landslide (Figure 5d).

### 3.3. UAV Data Results

As the field mapping was carried out in an emergency (in only 1 day, 14 March 2018 by Croatian geological Survey), the detailed orthophotos taken by UAV on the same day proved to be a valuable source for follow-up landslide mapping [12,41,42]. The landslide area, features, and damages can be clearly distinguished in these photos, i.e., cm size features could be mapped (Figure 6a). For the more complete landslide area interpretation, a 3D landslide area spatial model was also developed (Figure 6b).

### 3.4. LIDAR Based Data Analysis

**Figure 7:**
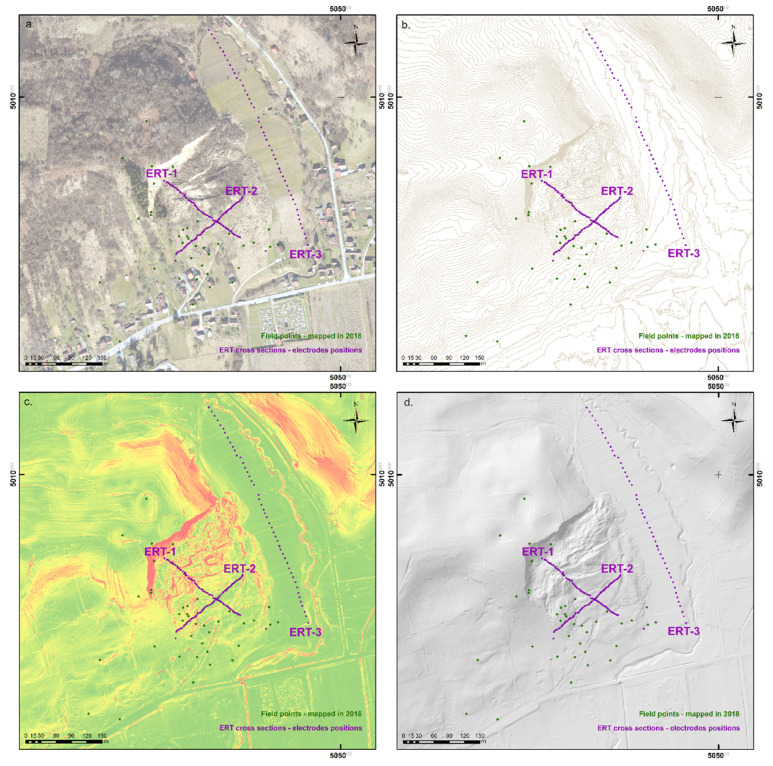
High resolution data from the Hrvatska Kostajnica area and landslide study area with mapped field points from 2018 and locations of ERT cross sections from 2020 and 2021: (**a**) detailed orthohpto from 2021; (**b**) 1 m contour lines based topography map; (**c**) detailed slope model; and (**d**) detailed terrain hillshade model.

**Figure 8:**
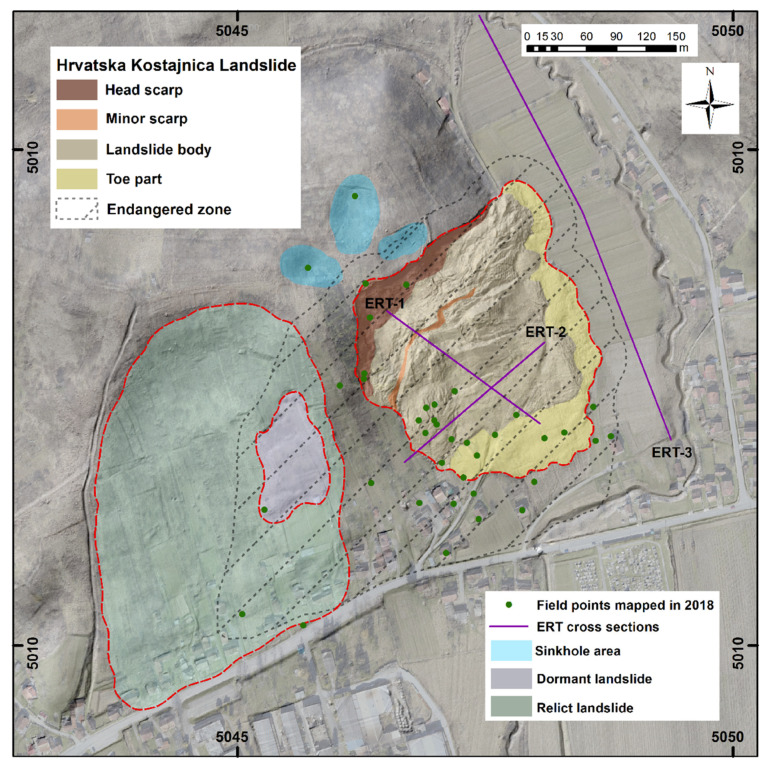
New Hrvatska Kostajnica Landslide Map with marked areas of active, dormant and relict landslide. Landslide areas are depicted with red dashed lines. On the map re-defined area of landslide(s) endangered zone is also marked (grey dashed polygon area).

Based on aerial LIDAR scanning, high resolution remote sensing data sets (Figure 7a–d) were used in landslide model development [6,43,44,45]. With the combination of a detailed orthophoto (Figure 7a), 1 m contour lines developed from LIDAR data (Figure 7b), a detailed slope model (Figure 7c), and terrain hillshade model (Figure 7d), quality terrain landslide surface model development (2D) was possible (Figure 8) [12,46]. These high resolution data were cross checked with acquired mapped data from 2018 and ERT data from 2020 and 2021. For the 3D landslide model development UAV model from 2018 (Figure 6b), cross sections from detailed DEMs and ERT measurements (cross sections) were analyzed and interpreted (Figures 10 and 11).

Using detailed LIDAR based data, precise terrain landslide surface model development was possible, which served as baseline for new landslide map development [47]. A precise terrain landslide surface model was also used for landslide features re-evaluation and mapping and the re-evaluation of possible endangered zones. As a result, the New Hrvatska Kostajnica Landslide Map was developed (Figure 8). The map was also field verified in September 2021 (within GeoTwinn project, with experts from the Geological Survey of Denmark and Greenland, GEUS).

### 3.5. Geophysical Measurements—ERT Data Interpretation

ERT has been applied to the area of research in order to investigate: the lithostratigraphic sequences, paleo-relief depth and morphology, the geometry of landslide body, the occurrence of new cracks (that could indicate an early-stage formation of new landslide), and the presence of weakened zones/fault areas [48].

Resistivity measurements are made by injecting a controlled current into the ground through two steel electrodes and measuring the potential drop at other two electrodes. Different electrode arrays, such as Wenner, Wenner–Schlumberger, dipole–dipole, etc., can be used for ERT surveys [49]. The Wenner–Schlumberger array was used in the research as it has a good signal response, the ability to resolve horizontal and vertical structures relatively well, and a greater penetration depth [50,51,52,53,54]. Field measurements in the presented study were performed using the POLARES 2.0 electrical imaging system (P.A.S.I. srl).

**Figure 9:**
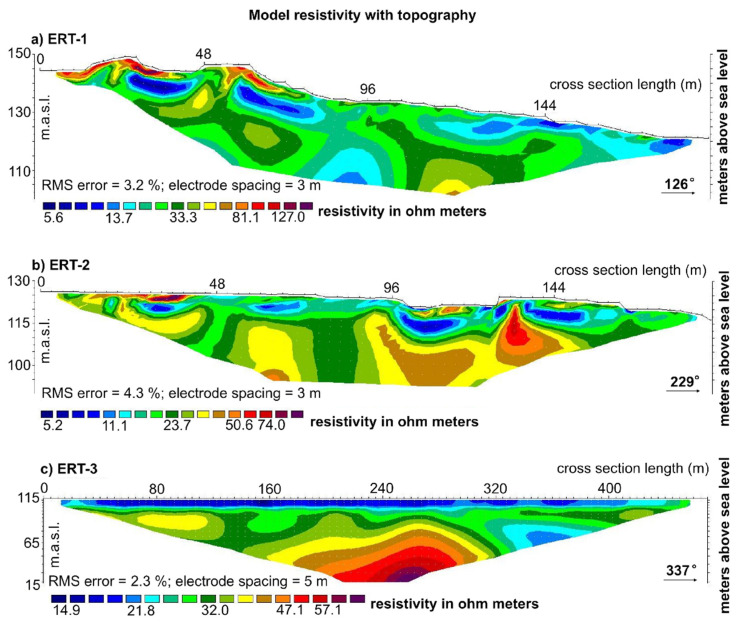
Electrical resistivity tomography results: (**a**) ERT-1 in the landslide body, cross section is oriented along the movement direction; (**b**) ERT-2 in the landslide body, cross section is oriented vertically on the movement direction; and (**c**) ERT-3 in the vicinity of the landslide, cross section is located in the “undisturbed soils”. On the ERT-1 and ERT-2 highly deformed/disturbed near surface areas can be depicted while on ERT-3 paleo-relief can be interpreted.

**Figure 10:**
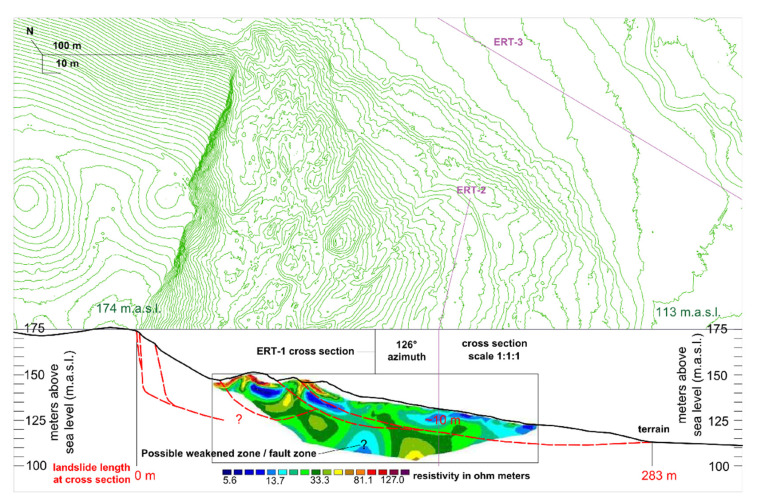
Hrvatska Kostajnica landslide cross section (parallel to movement) and interpreted ERT-1 data. At cross section area the length of the landslide is 283 m with multiple slide surfaces. The depth of the interpreted sliding surfaces varies from ~20–30 m at head scarp area, to ~10–20 m at landslide body area and ~1–10 m at landslide toe part area. In the middle part of the cross section possible fault zone is present (marked with question mark). The contour line interval on the topographical map is 1 m.

**Figure 11:**
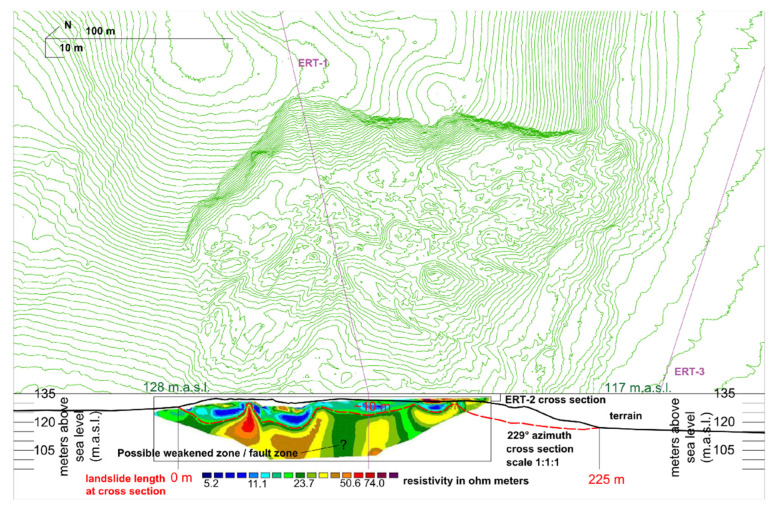
Hrvatska Kostajnica landslide cross section (perpendicular to movement) and interpreted ERT-2 data. At cross section area the length of the landslide is 225 m with slide surface at the depth of ~10 m. In the middle part of the cross section possible fault zone is present (marked with question mark). The contour line interval on the topographical map is 1 m.

In this study, three cross sections were carried out for the ERT survey and the positions of the electrodes/three ERT cross sections are shown in Figure 8, Figure 9, Figure 10 and Figure 11.

ERT-1 and ERT-2 were measured in the landslide body in October 2020, while ERT-3 was measured in the immediate vicinity of the landslide, in the Quaternary sediments in May 2021. Surveys were conducted using the Wenner–Schlumberger array for ERT-1, ERT-2, and ERT-3 at a frequency of 7.15 Hz and a maximum phase of 200 between the voltage signal and the current signal. The ERT-1 and ERT-2 were measured with 64 steel electrodes and the length of the cross sections was 189 m with an exploration depth of about 40 m. ERT-3 was measured with 48 electrodes and the length of the cross sections was 470 m with an exploration depth of about 95 m.

The apparent electrical resistivity data were inverted using the RES2DINV software [50] to obtain the 2D electrical resistivity images of the subsurface model [55,56,57,58]. The ERT-1 and ERT-2 were measured in a landslide body, and therefore the topography effect was taken into account during the execution of the data set inversion in RES2DINV software. The optimization method adjusts the 2D electrical resistivity model trying to iteratively reduce the difference between the calculated and measured apparent resistivity values [59]. The root-mean-squared (RMS) error provides a measurement of this difference. All three cross sections have acceptable RMS values: for ERT-1 RMS, 3.2%; for ERT-2 RMS, 4.3%; and for ERT-3 RMS, 2.3%. The 2D model sections obtained from data inversion are presented as resistivity tomograms (Figure 9a–c).

The tomograms measured in the landslide body (ERT-1 and ERT-2) show the variation of modelled electrical resistivity in-depth and along the line of investigation. ERT-1 and ERT-2 show a distinctly heterogeneous structure, both laterally and vertically, especially near the surface, but this was expected due to landslide presence and highly deformed/disturbed areas. There are low and high resistivity zones in these sections, but in general the values of resistivity are low. The aim of measuring these cross sections (ERT-1 and ERT-2) was to establish the depth(s) of sliding zone(s). The ERT-3 was measured outside the landslide zone in the undisturbed area/sediments/Quaternary deposits and consequently the variation of modelled electrical resistivity is less expressed. The aim of measuring this cross section (ERT-3) was to establish the “base resistance values of the undisturbed soil”.

From the detailed LIDAR data, detailed terrain/landslide cross sections were developed, and the ERT measurements conducted on these cross sections were interpreted as first step towards Hrvatska Kostajnica landslide model development (Figure 10 and Figure 11). Measured ERT-1 and ERT-2 length is 190 m (in field) while the cross-section length with data for interpretation is ~180 m with a depth of ~35 m.

On the ERT-1 and ERT-2, measured in the landslide body, where the material is deformed, the near surface variations in resistivity are more expressed, but still for the colluvial material is generally in the range of 5–20 Ohm meters. Somewhat different but still relatively low resistance values for ERT-1 and ERT-2 in the “bedrock” can be explained by the lithology and “interlayering” of the material.

In the vicinity of the landslide, the ERT-3 resistance values from 5 to 25 Ohm meters represent (mainly) saturated deposits made of clay, silt, and gravel. The thickness of these deposits are ~12 m (Quaternary deposits, Alluvial). Below that, the paleo-relief surface can be interpreted at the depth ~50–65 m. In the zone between the alluvial deposits and paleo-relief, surface resistance values vary from 25 to 50 Ohm meters. This “zone” is probably represented with Quaternary slope deposits in the upper part of the section and with the weathered interlayered materials in the lower part of the section (lower Sarmatian). Below the slope deposits and weathered zone, bedrock is expected (upper Badenian and lower Sarmatian) with resistivity values >50 Ohm meters (as for ERT-1 and ERT-2).

If we compare the resistance measurements of the “bedrock” on the ERT-3 with the ERT-1 and ERT-2 measured in the landslide body, we can conclude that “bedrock” is the same, but there are probably variations in the depth of the weathered zone and in the heterogeneity of the structure, i.e., in the number and thickness of “interlayers” (Figure 9a–c).

Moreover, it should be noted that the resistivity values of landslide location and bedrock slightly vary from the standard values in the literature, which is due to the different noise of data, saturation conditions, weathering effects, and fracturing of rock and minerals contained in the rock and soils [60].

## 4. Discussion

### 4.1. “Geo” and Laboratory Data Analysis

From the available geological and landslide data, it can be concluded that the area is generally prone to landslides (see Section 2.3. and Figure 4) [27,28]. The developed 26 m high detailed geological column at main scarp of Hrvatska Kostajnica landslide reveals that the landslide occurred mainly in the “marly” sediments, but clayey (weathered) limestone is also present at location [24]. The landslide affected the soil layer, weathered zone, and “bedrock”, but no clear sliding zone(s) was mapped on the field during geological column development and during engineering geological mapping in 2018 [25]. The assumption is that there were multiple weakened “zones” due to rhythmic changes within material, i.e., geological heterogeneity. Multiple field measurements and samples from the landslide head scarp were taken for the determination of on-site material characteristics and laboratory analysis. Field pocket penetrometer values and CaCO_3_ ratio variations also indicate differences in material properties (Figure 4d). Within weathered clayey limestones on bedding plains and fracture planes, “preferred” seepage and water “leakage” could have occurred, and at the same time in these discontinuities thin clay layer(s) are sometimes present. Moreover, seven interchanges of marls–clayey limestone can be tracked at geological column/landslide main scarp (Figure 4). On this geological column, the main geo-specific characteristics of the location can be observed: the material is mainly marl with silts and it is interlayered with clayey limestone. This “interlayering” is common, and the materials are generally weathered. Moreover, the bulk analysis of marls shows that the main mineral components are calcite and clay minerals (i.e., smectite, ilite, kaolinite, vermiculite), with some of them with high shrink-swell capacity [24]. All the “geo” data indicate multiple sliding surfaces as result of “rhythmic” changes within the material.

### 4.2. DInSAR Data Analysis

DInSAR was applied to detect the pre-failure behavior of the slope, to detect the sudden surface displacements caused by landslide, and to detect the deformations after the event. The results are summarized as follows [26] and supplemented in [30]: (i) A sudden large displacement of the landslide, which exceeds a half wave length of the SAR signal (in the case of Sentinel-1 it is 2.8 cm), could not be detected. This is essential according to the principle of DInSAR [31]. (ii) The displacement, which continuously and gradually changes, could be detected [30,32]. It was found that the displacement gradually increased/decreased in the area of the active landslide slope during one year before the slope collapse. As this change is “rhythmic”, it is not conclusive from the deformation development viewpoint. These “seasonal changes” are more expressed in the wider areas near Una River (500 m from the landslide). (iii) Even though the DInSAR analysis did not reveal any definite long term slow changes on the landslide area before or after the landslide event, it is still worthwhile to study an effective method for the application of DInSAR in the slope failure research(es) [26,30]. (iv) The undertaken DInSAR data analysis was used in surface displacement behavior trend determination and it indicates that the landslide was a “short and extreme” event rather than a long-term, ongoing, slow slope deformation process as there are no definite long-term slow changes on the landslide area before or after the landslide event. That is in accordance with assumption about landslide triggering factors: extreme on-site climate conditions triggered Hrvatska Kostajnica landslide.

### 4.3. Orthophotos and UAV Data Analysis

From the available historical orthophotos, UAV footage and developed 3D model the following can be concluded [12,33]: (i) from the 1960-ies the area (partially) developed from arable land to settlements; (ii) the destroyed, damaged and endangered objects can be identified on these data sets; (iii) the detailed orthophotos and the developed 3D model were a great asset in landslide features mapping. Additionally, as an indirect conclusion, the following assumption can be made: even though that anthropogenic factor can be decisive in landslide triggering, due to the characteristics of this landslide event (deep seated with multiple sliding zones), anthropogenic factors played no or only a minor role in this case.

### 4.4. New Landslide Map Development

On the New Hrvatska Kostajnica Landslide Map, all the areas which are/or could be influenced by landslide(s) are reviewed and as a result the area of Endangered zone is redefined [43,45,46]. Within the Hrvatska Kostajnica landslide body, there is still activity: the erosion of the head scarp area due to atmospheric influences is an ongoing process i.e., the landslide is developing retrogressively [6,44,47]. Moreover, the settlement of the colluvium at the toe part area is ongoing at some (minor) rate. In the landslide body, there are still many wide-open cracks which are not stabilized. In the case of extreme rain, new movements within landslide body are possible. That is the reason for the endangered zone encircling the area of the active landslide body (same as on field map and report from 2018), but after revision of the available data the endangered zone was enlarged and an area with no traces of deformations was included with the area of the dormant landslide. The reason for that is the following: according to the data analysis, it became evident that to the east a relict landslide area exists (already partially mapped on the field in 2018), and within that landslide body a dormant landslide can also be distinguished (Figure 8). Between these landslides, a steep “terrain ramp” exists. It is approximately 75 m wide and 250 m long and without visible traces of deformation(s). However, is reasonable to expect for this (in this moment stable) area to eventually become un-stable and that the existing objects downslope could be endangered in the future by movements, i.e., new landslide(s). The new “terrain ramp” landslide(s) could also reactivate the dormant landslide to the east. Therefore, for ~20 households, landslide risk still exists in some, not negligible, percentage. On the map three sinkhole areas in the vicinity of the landslide head scarp are also marked. Their “importance” is in the “channeling” of the surface waters (rain) into the landslide body and existing terrain ramp area. The positions of the ERT cross sections are also marked on the map: ERT-1 and ERT-2 in the landslide body and ERT-3 in the vicinity of the landslide

### 4.5. ERT Data and Initial Landslide Model Development

ERT-1 is located in the central part of the landslide body, parallel to the mass movement (Figure 8 and Figure 10) and consists of layers with different resistance values varying from 5–130 Ohm meters [59]. The upper part of the cross section refers to unsaturated residual and saturated colluvial soil with different resistivity values (in the range of 5–20 Ohm meters) [40,55]. Very low resistance values in this layer are saturated colluvial soils/materials [48,59]. The thickness of these deposits (colluvial material) varies, but it is generally in the range of 10–20 m, i.e., it is thicker near the head scarp whereas it is thinner near the toe part of the landslide. Below colluvial materials, the resistivity values are higher but still relatively low, in the range of 20–50 Ohm meters. This can be explained by material properties, i.e., the sediment is represented by weathered deposits which are built of marls with interlayers of clayey limestones, calcareous marls, and silty marls (see geological column, Figure 4). Even some weakened zones/fault areas can be assumed on this cross section (middle part, Figure 10). Higher resistivity values (>50 Ohm meters) are found on the deepest part of the cross section, indicating that the materials are generally still the same but the weathering effect is probably not so expressed (bedrock; upper Badenian and lower Sarmatian).

ERT-2 is located in the central part of the landslide body and is perpendicular to the ERT-1 and mass movement (Figure 8 and Figure 11) and consists of layers with different resistance values varying from 5–80 Ohm meters [59]. As for ERT-1, for ERT-2, the upper part of the cross section refers to unsaturated residual and saturated colluvial soil with different resistivity values (in the range of 5–20 Ohm meters) [40,55]. Very low resistance values in this layer are saturated colluvial soils/materials [48,59]. The thickness of these deposits (colluvial material) varies, but it is generally in the range of around 10 m, i.e., it is thicker near more “deformed” areas in the landslide body whereas it is thinner near toe part of the landslide. On the ERT-2, the “deepest” slip surface can be interpreted with relatively high accuracy (Figure 11). As for ERT-1, on the ERT-2, below colluvial materials, the resistivity values are higher but still relatively low, in the range of 20–50 Ohm meters, as the materials are the same: weathered deposits which are built of marls with interlayers of clayey limestones, calcareous marls, and silty marls. The weakened zone/fault area which can be assumed on the ERT-1 also can be interpreted on ERT-2 (middle part). As for ERT-1, higher resistivity values (>50 Ohm meters) for ERT-2 are found on the deepest parts of the cross section, indicating that the materials are generally still the same but the weathering effect is probably not so expressed (bedrock; upper Badenian and lower Sarmatian).

ERT was applied on the Hrvatska Kostajnica landslide to determine the physical properties of soil and rock, subsurface lithology, groundwater condition and geometry of the slide surface [11,40,48,55,60]. According to the measured resistances, it can be concluded that the landslide has multiple sliding surfaces and that the highly deformed materials are present mainly in the main body of the landslide (Figure 10 and Figure 11). The cross sections within landslide body (ERT-1 and ERT-2) revealed the zones with the colluvial materials, in the range of 5–20 Ohm meters, in the upper part of the cross sections and possible weakened zones (faults) in the lower part of the cross sections. Below colluvial materials, weathered and interlayered materials are present (with resistivity values in the range of 20–50 Ohm meters). The “bedrock” on the ERT-1 and ERT-2 is probably a heterogeneous structure (interlayered materials), but not so weathered and with somewhat higher resistivity values (>50 Ohm meters). The cross section in the vicinity of the landslide body (ERT-3) revealed that the near surface part of the cross section refers to (mainly) saturated soils with low resistivity values in the range of 5–25 Ohm meters. Below that, a paleo-relief can be interpreted with resistance values from 25 to 50 Ohm meters and the bedrock is expected below the paleo-relief with resistivity values >50 Ohm meters (same as for ERT-1 and ERT-2).

### 4.6. Multi-Level Sensing Data Combination Benefits

With multi-level sensing technologies, multiple data sets have been acquired (historical orthophotos, UAV, DInSAR, LIDAR, and ERT data) and every analyzed different data set has given a different usable conclusion, which were used all-together in order to develop the landslide model.

Orthophotos provided insight into otherwise unavailable historical data about land use. Detailed UAV data helped greatly in landslide features mapping. DInSAR analysis indirectly pointed out that on the landslide area there are no slow and long-term deformations ongoing. LIDAR data provided the basis for the new landslide map and cross sections. ERT data provided insight into material properties beneath the surface (3D). As each of the used data sets has its benefits (but also weak points as they are mostly 2D data), by combining them, the first step landslide model was developed. Still, for the better model, more detailed 3D data are needed (e.g., borehole data in order to verify the interpreted sliding surface(s), materials depth and composition; the boreholes can be dimensioned based on the presented model), and the data concerning climate conditions should be taken into consideration if the landslide model is intended to be used in forecasting.

The initial developed landslide model indicates a landslide area of ~5 ha with multiple sliding surfaces, the deepest at ~30 m, but generally in the landslide body around ~10–20 m of depth. As the landslide endangered zone is relatively large, ~12.5 ha, and in this area ~15 households still exist, further continuous monitoring is also recommended. 

As for the question: Can it happen again? The answer is: Yes, it can. Probably not in the same scale (not so large landslide as in 2018), but if the extreme climate conditions in relatively a short period (couple of days) occur again, there is a possibility for a new landslide(s) to occur in a previously undisturbed area and there is also a possibility for a re-activation of “old” landslides that could endanger properties and create risk for residents.

## 5. Conclusions

The landslide in Hrvatska Kostajnica (with area of ~300 × 300 m and maximum of ~60 m height difference), Croatia was activated on March 13 2018 after rapid temperature change and snowmelt (~80 cm of snow cover melted). At the same time, Una River was flooding (~400 m from landslide toe part and with ~5 m higher water level than usual). The landslide completely destroyed and heavily damaged multiple households (~10) and lives were endangered.

Due to relatively sparse data immediately after the landslide event, field mapping and UAV data collection were performed with the revision of available geological and remote sensing data. For the landslide, a new geological column was developed at the head scarp area (~26 m of column/head scarp height) with material sampling and laboratory testing. Moreover, available remote sensing data were analyzed, namely satellite images and historical orthophotos.

Main new insightsabout Hrvatska Kostajnica landslide were gained by LIDAR based and geophysical data analysis. From detailed LIDAR-based data, a precise terrain landslide surface model was developed and used for the cross section and new landslide map development. On the New Hrvatska Kostajnica Landslide Map, the areas of dormant and relict landslides in the vicinity are also marked and the area of endangered zone is re-defined (Figure 8). The conducted ERT measurements were interpreted as a first step towards Hrvatska Kostajnica detailed landslide model development: they provided insight into the slide surface depths and complexity of this landslide (cross sections ERT-1 and ERT-2). Cross section ERT-3 in the landslide vicinity provided reference values for the “undisturbed saturated soils”.

As every (developed) model is simplified in some way, so is here presented “first step” Hrvatska Kostajnica landslide model, but projected sliding surface depths, landslide area and endangered zone are still defined. As the data about climate conditions were not taken into consideration, further research should be an on-going process in order to make the landslide model more detailed, reliable, and usable for the local community in terms of forecasting and early warning. Still, the New Hrvatska Kostajnica Landslide Map with marked areas of active, dormant, and relict landslides and the re-defined area of endangered zone is already in the form which the local community can use in landslide risk assessment and urban planning.

## Figures and Tables

**Table 1:** List of reviewed and analysed main data sets for the study area i.e., landslide in Hrvatska Kostajnica.

Data Type	Short Description
Maps, geological column, landslide inventory	Small scale Landslide susceptibility map of Croatia (LSMC CGS, PODOLSZKI et al., 2015) [27]
	1:500,000 Engineering geological map of Yugoslavia (ČUBRILOVIĆ et al., 1967) [28]
	1:300,000 Re-classified lithological map of Croatia (internal CGS)
	1:100,000 Basic geological map—sheet Kostajnica with Guide (JOVANOVIĆ and MAGAŠ, 1986a; 1986b) [23]
	1:100,000 Geological map of Sisačko-Moslovačka County (CGS, 2014) [29]
	1:100,000 Landslide susceptibility map of Sisačko-Moslovačka County (safEarth project, CGS; BOSTJANČIĆ et al., 2021) [4]
	1:5000 Geological map for Hrvatska Kostajnica landslide—in progress (internal CGS, planned for 2022, field work conducted)
	1:5000 Topographic map (National Geodetic Administration of Croatia, NGA)
	1:5000 Engineering geological map for Hrvatska Kostajnica landslide area—field mapping conducted in 2018 (CGS) [25], map data up-dated and verified in 2021 within GeoTwinn project with Geological Survey of Denmark and Greenland (CGS and GEUS)—results presented in this paper
	Geological column of Hrvatska Kostajnica Landslide (modified after GRIZELJ et al., 2020) [24]
	Developed Landslide inventory for study area (internal CGS 2018); up-dated and reviewed in 2021 (CGS and GEUS)
Remote sensing images	Satellite images—SAR data from Sentinel-1 a and B satellite: 150 scenes for the period of 1 December 2014–13 June 2020 (European Space Agency, ESA)
	Orthophotos from 1968 (National Geodetic Administration of Croatia, NGA)
	Orthophotos from 2014–2016 (National Geodetic Administration of Croatia, NGA)
	Orthophotos from 2017 and 2018 (National Geodetic Administration of Croatia, NGA)
	Orthophotos from 2020 (National Geodetic Administration of Croatia, NGA)
	UAV data—Orthophotos from 2018 with 5 × 5 cm pixel size(CGS)
	UAV data—developed 3D landslide area spatial model from 2018 (CGS)
LIDAR with developed DEMs and data for Hrvatska Kostajnica	Airborne LIDAR scan, early spring 2021, 20 points per m^2^ (Flycom Technologies for RESPONSa project, CGS)
	0.5 × 0.5 m cell size DSM—digital surface model (developed from LIDAR data, Flycom and CGS)
	0.5 × 0.5 m cell size DTM—digital terrain model (developed from LIDAR data, Flycom and CGS)
	0.5 × 0.5 m cell size DTMh—digital terrain model hillshade (developed from LIDAR data, CGS)
	0.5 × 0.5 m cell size DTMs—digital terrain model slope (developed from LIDAR data, CGS)
	1 m contour line topography map model (developed from LIDAR data, CGS)
Field point data and cross section data	32 field points (geological and engineering geological) in landslide area (CGS 2018)
	6 field points (engineering geological) for wider area (CGS 2018)
	ERT measurements in the landslide body: 2 cross sections in 2020 (CGS)
	ERT measurements in the vicinity of the landslide area: 1 cross section in 2021 (CGS) and 1 cross section is planned for 2022 (CGS)
Laboratory data	Mineralogical and geochemical analysis included: X-Ray Powder Diffraction (XRPD analysis,6 samples), chemical analysis of major and trace elements (7 samples) and measurement of CaCO_3_ using Scheiblers calcimeter (15 samples) accompanied by paleontological analysis: calcareous nannofossil, palynological, foraminiferal and ostracod analysis (in detail supplemented in GRIZELJ et al., 2020) [24]

## Data Availability

Not applicable.
